# Impact of Epic Smartlist and Lumens Software in Improving OP-29 Compliance at a Tertiary Health Care Network

**DOI:** 10.7759/cureus.40193

**Published:** 2023-06-09

**Authors:** Zarian Prenatt, Hammad Liaquat, Troy Lovett, Joseph Evans, Manasa Srivilli, Nicholas Marzotto, Noel Martins

**Affiliations:** 1 Internal Medicine, St. Luke's University Health Network, Bethlehem, USA; 2 Gastroenterology, St. Luke's University Health Network, Bethlehem, USA; 3 Medical School, Lewis Katz School of Medicine at Temple, St. Luke's University Health Network, Bethlehem, USA; 4 Product Management - Epic Lumens, St. Luke’s University Health Network, Bethlehem, USA

**Keywords:** colon cancer surveillance, electronic health record (ehr), surveillance colonoscopy recommendations, op-29, epic lumens, quality improvement

## Abstract

Background

OP-29 is a Centers for Medicaid and Medicare Services (CMS) measure to ensure that endoscopists recommend appropriate follow-up intervals after normal colonoscopy in average risk patients. Failure to report OP-29 compliance can adversely affect hospital quality star rating as well as reimbursement for health care. The aim of our quality improvement project was to improve OP-29 compliance to the top decile over three years.

Methodology

Our sample included patients between 50-75 years of age who received average risk screening colonoscopies with normal findings. We provided intensive education to endoscopists about the importance of OP-29 compliance, developed an Epic Smartlist that directs our endoscopists to list an appropriate reason for colonoscopy intervals other than 10 years, and monitored OP-29 compliance monthly. We became the first health network in the United States to implement the Lumens endoscopy report writing software (Epic Systems Corporation, Verona, USA) and added the OP-29-related Epic Smartlist to the Lumens colonoscopy note template. All statistical analyses were conducted in SPSS version 26 (IBM Corp., Armonk, USA) to compute the means and frequencies of outcomes.

Results

Our sample included 2,171 patients with a mean age of 60.5 years of whom the majority were female (57.2%) and Caucasians (90%). Our OP-29 score increased from 87.47% to 100% over the course of three years, and this steady improvement was seen broadly across our network. We compared our network score averages to our state and national averages and consistently demonstrated higher compliance rates while reaching the top decile by 2020.

Conclusion

We believe our improved OP-29 compliance has reduced colonoscopy overutilization, improved health care quality, and reduced health care costs for our patients and health network. To our knowledge, this is the first reported project towards improving OP-29 compliance utilizing the Epic Lumens software. Epic Lumens (Epic Systems Corporation, Verona, USA) added this Smartlist as quick buttons in the standard colonoscopy procedure note templates they built for other organizations to improve health care quality and cost nationally.

## Introduction

The Hospital Outpatient Quality Reporting (OQR) Program is a pay-for-quality data reporting initiative that was mandated by the Tax Relief and Health Care Act of 2006 [1]. The Centers for Medicaid and Medicare Services (CMS) implemented this program, which requires hospitals to submit quality measures provided in the outpatient setting. CMS was granted authority by the Balanced Budget Act of 1997 to implement a prospective payment system for hospital outpatient services, known today as the Hospital Outpatient Prospective Payment System (OPPS) [2]. If hospitals fail to meet the Hospital OQR Program requirements, a penalty is applied to OPPS payments for applicable services, providing a financial incentive for hospitals to report their quality measures data. 

Payments for hospitals participating in the Hospital OQR Program are determined by specific measures, which identify processes of care, imaging efficiency patterns, care transitions, and patient safety [1]. CMS has identified specialty areas as having standard and frequent outpatient procedures performed, such as colonoscopies and other diagnostic procedures. One of the OQR measures is OP-29, which refers to the appropriate follow-up intervals for normal colonoscopy in average-risk patients. Poor OP-29 compliance can adversely affect hospital quality star rating and reimbursement for health care [2]. Our OP-29 score at St. Luke’s University Health Network (SLUHN) was 87.47% in 2017 first quarter (Q1). This was slightly above the Pennsylvania/New Jersey average and the national average, but not in the top decile. The aim of our project was to improve OP-29 compliance to the top decile over the course of three years using an Epic Smartlist (Epic Systems Corporation, Verona, USA) in addition to providing intensive education and feedback to endoscopists. 

This article was previously presented as a meeting abstract at the 2021 Digestive Disease Week (DDW) Annual Scientific Meeting on May 21, 2021 [3].

## Materials and methods

The study took place from January 2017 to March 2020 within our 14-hospital network located in Eastern Pennsylvania and New Jersey. We only included data from the seven hospitals that were part of the network when the study began in 2017. The study was approved by the St. Luke's University Health Network Institutional Review Board (#2020-111). We included all patients between 50-75 years of age who received average-risk screening colonoscopies with normal findings according to the United States Multi-Society Task Force for Colorectal Cancer guidelines. We excluded patients given a follow-up interval of 10 years and those given a follow-up interval other than 10 years for whom a reason was given, such as no adenoma with age > 66 years; life expectancy < 10 years; personal/family history of colon polyps or colon cancer; or comorbidities significantly increasing complication risk from further procedures.

**Figure 1:**
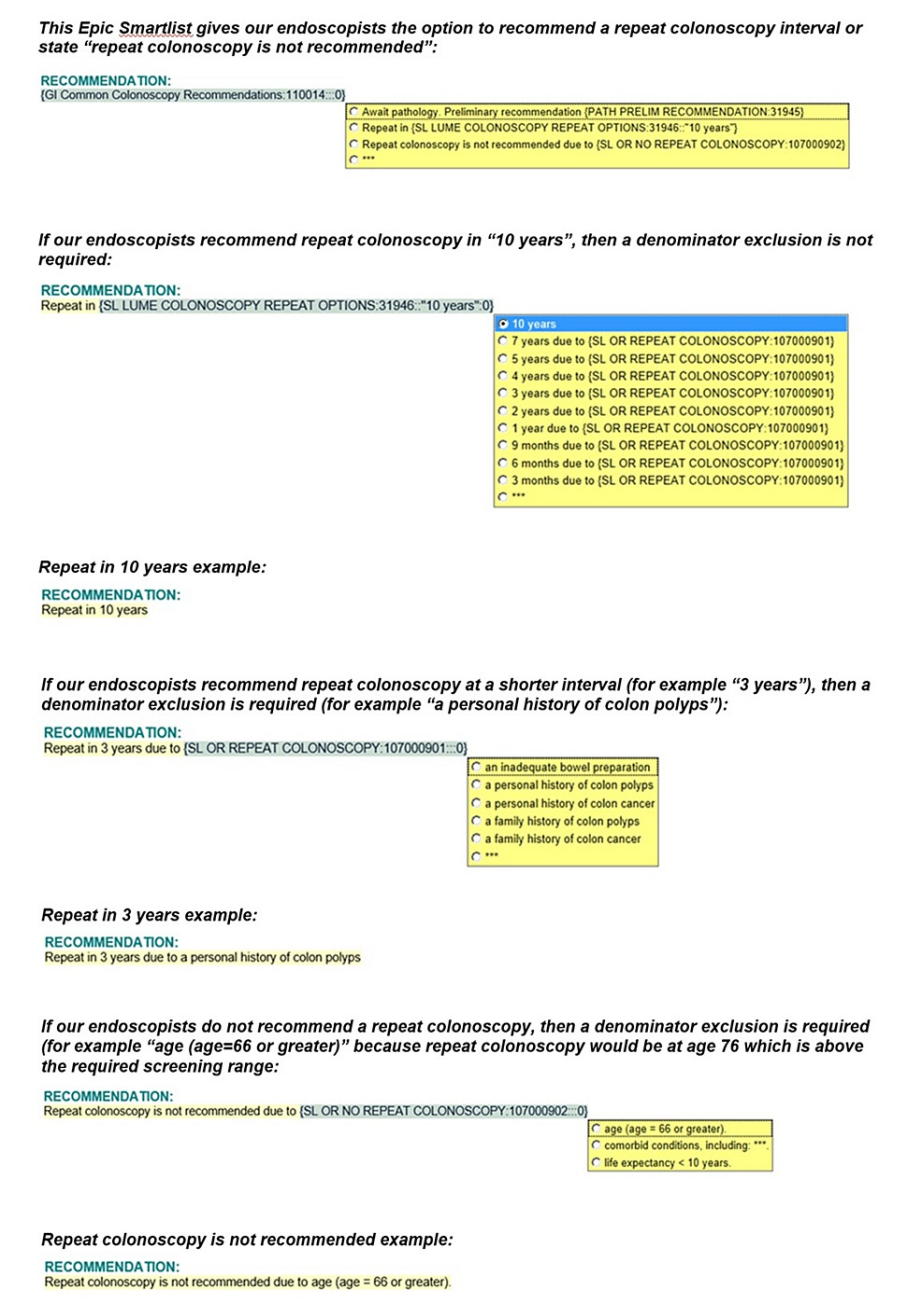
Epic Smartlist demonstrating the available options to choose from regarding repeat colonoscopy interval

This was a prospective quality-improvement initiative that sought to improve OP-29 compliance through various interventions following the Plan-Do-Study-Act model. We first obtained the baseline OP-29 scores, identified barriers to compliance, and provided intensive education to endoscopists about the importance of OP-29 during department meetings. We then developed and implemented an Epic Smartlist that directed endoscopists to recommend a repeat colonoscopy interval and list an appropriate reason if the interval is not 10 years (Figure 1).

In May 2019, SLUHN became the first network in the United States, and the second in the world, to implement the Lumens endoscopy report writing software. We added the OP-29-related Epic Smartlist to the Lumens colonoscopy procedure note template. Our clinical analytics team monitored the OP-29 compliance monthly at both the network and individual hospital levels, and we provided regular feedback to endoscopists as needed. When endoscopists were not compliant with OP-29, they were contacted via email or phone call to discuss those specific instances and re-educate them about the importance of OP-29 compliance. All statistical analyses were conducted in SPSS version 26 (IBM Corp., Armonk, USA) to compute the means and frequencies of outcomes. 

## Results

**Figure 2:**
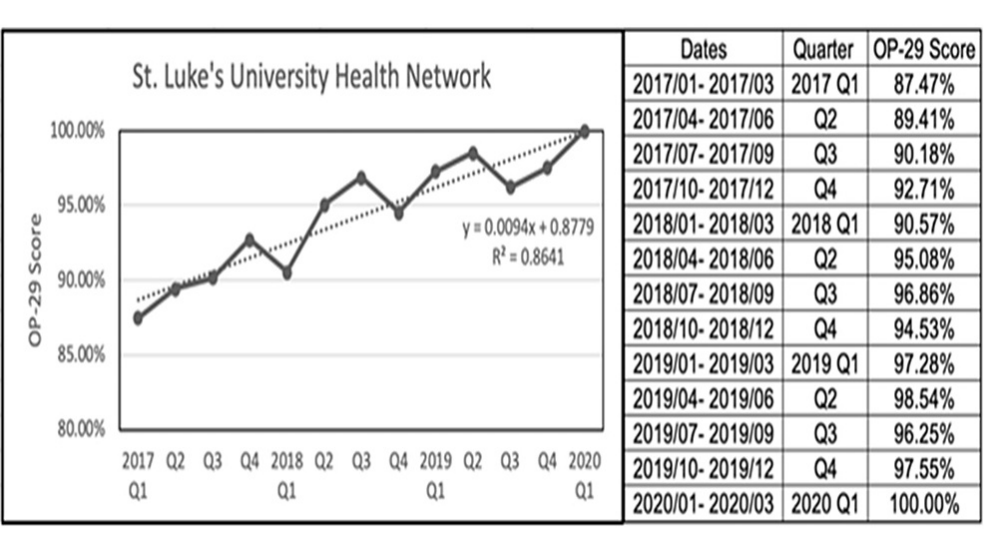
SLUHN quarterly OP-29 scores from 2017 Q1 to 2020 Q1 SLUHN: St. Luke’s University Health Network

**Figure 3:**
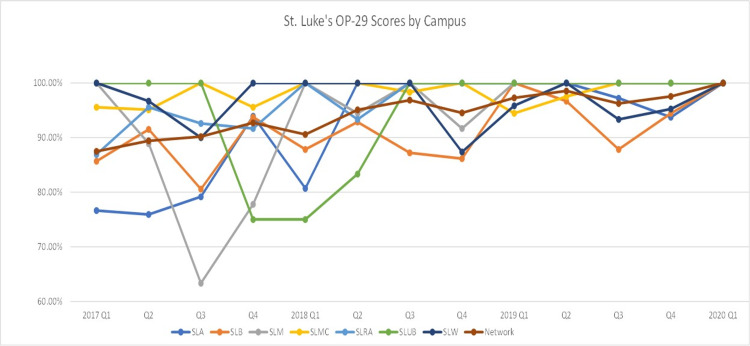
Quarterly OP-29 scores from 2017 Q1 to 2020 by Campus SLA: St. Luke’s Allentown SLB: St. Luke’s Bethlehem SLM: St. Luke’s Miners SLMC: St. Luke's Monroe Campus SLRA: St. Luke's Anderson SLUB: St. Luke’s Upper Bucks SLW: St. Luke’s Warren Network: Average across the St. Luke’s campuses

**Figure 4:**
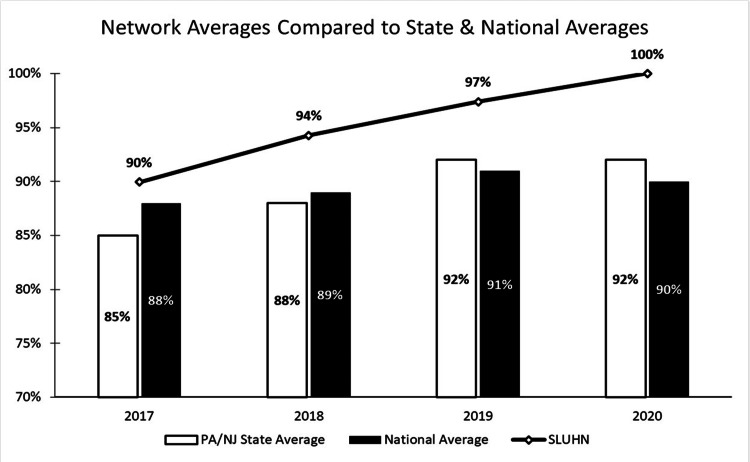
SLUHN averages compared to state and national averages SLUHN: St. Luke’s University Health Network; PA: Pennsylvania; NJ: New Jersey

Our sample included 2,171 patients with a mean age of 60.5 years of whom the majority were female (57.2%) and Caucasians (90%). Our quality improvement project spanned the course of three years with analysis of OP-29 compliance conducted from January 2017 Q1 to March 2020 Q1. Our OP-29 score increased from 87.47% in 2017 Q1 to 100% in 2020 Q1 (Figure 2). We extracted data from seven of our campuses, and the steady improvement in compliance scores was seen broadly across our network (Figure 3). We also compared our network score averages to our state and national averages (Figure 4), and we consistently demonstrated higher compliance rates while eventually reaching the top decile in 2020. 

## Discussion

Endoscopists are expected to provide a follow-up recommendation after a colonoscopy, which is an extremely common clinical scenario requiring guidance [4]. The United States Multi-Society Task Force for Colorectal Cancer guidelines for average-risk patients who undergo a high-quality colonoscopy with normal findings recommend a repeat screening colonoscopy in 10 years [4]. This recommendation is supported by data showing that individuals with a normal colonoscopy are at lower risk for colorectal cancer [5-7]. With that said, repeating colonoscopies sooner than recommended can increase health care costs and the risk of complications without increasing patient benefit [8,9].

Numerous studies have identified the overuse of colonoscopy as measured by physician recommendations [9-11]. Additional studies have also demonstrated that there is no benefit to repeating screening or surveillance colonoscopy earlier than recommended [8]. Some proposed explanations for deviation from interval guidelines include fear of missing cancer, liability concerns, or belief that performing additional interventions is in the best interest of the patient’s health. Our study indicates that continuous feedback, education, and implementation of an electronic health record (EHR) endoscopy smart set are effective interventions to address these issues and improve adherence to interval guidelines. 

Health information technology (HIT) has become fundamental to healthcare and is a primary strategy for not only improving the quality of care and optimizing patient management but also enhancing the capability to perform large-scale research and identify effective interventions [12-14]. Commercial EHR systems are crucial because of their ability to produce data required to qualify for financial incentives and avoid penalties for non-compliance. Epic Systems was founded in 1979 and has become a main provider of EHR software for large and medium-sized organizations. Epic has built an integrated platform for almost all areas of care, and there are numerous Epic modules that have been released for specific areas [15]. 

Epic Lumens is the reporting module for endoscopy, which provides tools for viewing and managing endoscopy images sent to Epic by external endoscopy systems [15]. To our knowledge, this is the first reported project towards improving OP-29 compliance, which utilized the Epic Lumens software. SLUHN became the first network in the United States to implement the Lumens endoscopy reporting software in May 2019. We observed a 100% OP-29 compliance rate at SLUHN the following year in 2020, and we believe a large component of this success can be attributed to the integration of the Lumens software with the OP-29 Smartlist. With the development of the Epic Lumens endoscopy reporting software and the improved OP-29 compliance we observed in our study, we believe it is important for large-scale healthcare delivery systems to obtain the most up-to-date advances in EHR technology to optimize patient care, data collection, and workflow efficiency. 

Throughout the course of our quality improvement project, we observed a few ongoing challenges. Our clinical analytics team discovered that not all endoscopists were using the Epic Smartlist. Additionally, some endoscopists were not using the Epic Smartlist for all colonoscopies. Given that the endoscopy reporting software was a new addition during our study, this nonadherence could be attributed to the natural difficulties that arise when adjusting to a new tool. These difficulties include learning how to utilize the Smartlist and incorporating it into one’s typical EHR workflow. Another explanation could be that the endoscopists were simply unaware of the Smartlist or were forgetting to utilize it. We also observed that compliance was lower among the non-gastroenterologists and non-employed SLUHN endoscopists. We suspect the lower compliance rate among these groups was due to the limited reach of our education regarding OP-29. The non-gastroenterologists and non-employed SLUHN endoscopists were not a primary target for our intensive education, and thus, were not as informed as our SLUHN endoscopists. We addressed these adherence challenges through additional education and by adding the Epic Smartlist to the Epic Lumens colonoscopy template. This increased our OP-29 compliance rate to 100%, but we anticipate continued intermittent non-adherence and plan to continue to provide regular feedback and training.

Our study demonstrates that EHRs possess the capability to improve the quality of care and safety in health care, and these advancements can occur once providers understand and adapt to the new functions consistently and effectively. 

## Conclusions

Our project sought to improve OP-29 compliance at SLUHN over the course of three years. We observed a successful improvement with compliance scores increasing from 87.47% in 2017 to 100% by the end of our study in 2020. We believe our improved OP-29 compliance has led to reduced colonoscopy overutilization, improved healthcare quality, and reduced health care costs for our patients and our health network. Our top decile OP-29 compliance is one of many factors that has contributed to our attainment of a five-star hospital quality rating. 
